# Influenza Viruses: Innate Immunity and mRNA Vaccines

**DOI:** 10.3389/fimmu.2021.710647

**Published:** 2021-08-31

**Authors:** SangJoon Lee, Jin-Hyeob Ryu

**Affiliations:** ^1^Department of Infection Biology, Faculty of Medicine, University of Tsukuba, Tsukuba, Japan; ^2^BIORCHESTRA Co., Ltd, Daejeon, South Korea; ^3^BIORCHESTRA Co., Ltd, Cambridge, MA, United States

**Keywords:** mRNA, vaccine, influenza virus, innate immunity, inflammasome, cytokines, inflammation

## Abstract

The innate immune system represents the first line of defense against influenza viruses, which cause severe inflammation of the respiratory tract and are responsible for more than 650,000 deaths annually worldwide. mRNA vaccines are promising alternatives to traditional vaccine approaches due to their safe dosing, low-cost manufacturing, rapid development capability, and high efficacy. In this review, we provide our current understanding of the innate immune response that uses pattern recognition receptors to detect and respond to mRNA vaccination. We also provide an overview of mRNA vaccines, and discuss the future directions and challenges in advancing this promising therapeutic approach.

## Introduction

The innate immune system serves as the first line of host immune response against pathogens but also virus-based vaccines containing either attenuated or inactivated viruses to prevent infectious diseases. After vaccination, the innate immune system identifies and removes vaccinated cells while coordinating the adaptive immune responses in the form of antigen-specific reactions, thereby sustaining long-term protection from the viral infection. To rapidly detect and defend against the various viruses, the immune cells have evolved to acquire multiple pattern-recognition receptors (PRRs) such as Toll-like receptors (TLRs), retinoic acid-inducible gene I (RIG-I)-like receptors (RLRs), and the nucleotide-binding oligomerization domain (NOD)-like receptor family proteins (NLRs) (1, 2). Through such receptors, the innate immune system is activated in a tightly regulated manner while retaining the adaptive immune response, but without excessive innate immune responses that can cause tissue damage and systemic inflammation, which are harmful to the host. In the present review, we discuss innate immune recognition, activation of inflammasome, and cytokine secretion in response to influenza virus infection and its mRNA vaccines. We also emphasize mRNA vaccines as promising tools for the prevention and control of influenza virus disease.

## Brief History on Influenza Vaccines to Date

**Figure 1:**
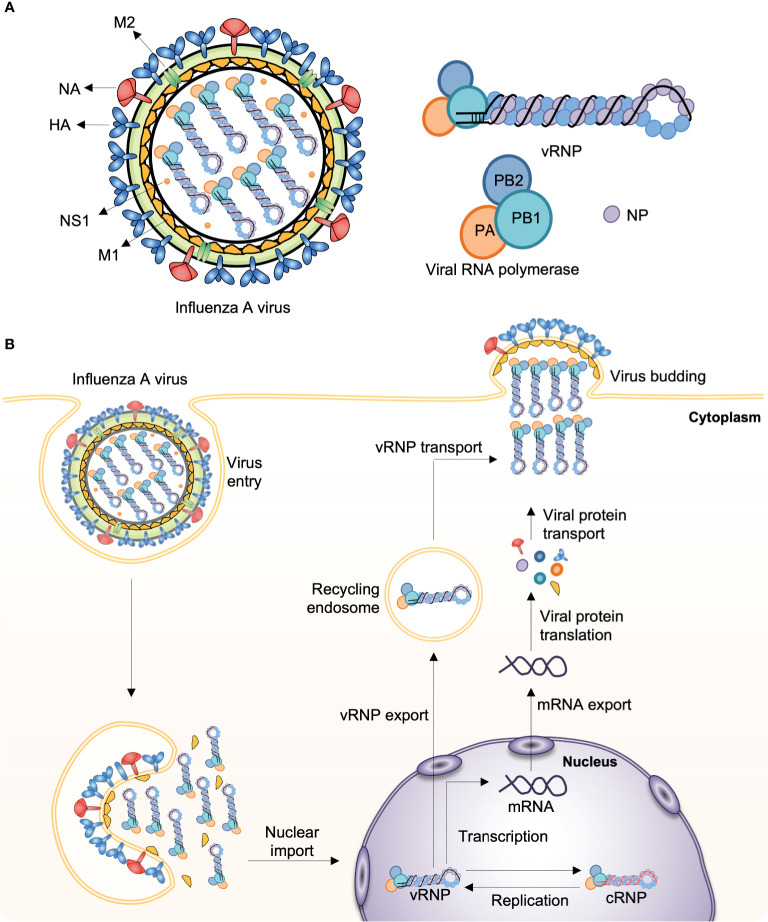
Influenza A virus structure and replication. **(A)** The influenza A virus (IAV) genome comprises eight segmented, and single- and negative-stranded RNAs (vRNA). Each segment is encapsulated by a nucleoprotein (NP) and, mediated by the RNA polymerase, they form the viral ribonucleoprotein (vRNP) complex (PB1, PB2, PA, and NP), which is the essential unit for both transcription and replication. **(B)** Infecting vRNP is transported into the nucleus where viral transcription and replication occurs, followed by progeny vRNP production. After the progeny vRNP is exported to the cytoplasm, the virus is assembled. HA, haemagglutinin; M1, matrix protein; M2, membrane protein; NA, neuraminidase; NS1, nonstructural protein 1; PA, polymerase acidic protein; PB1, polymerase basic protein 1; PB2, polymerase basic protein 2.

Influenza viruses cause some of the most virulent respiratory tract infections in humans. Seasonal influenza virus epidemics are estimated to lead to up to 290,000–650,000 deaths per year globally (3). Influenza viruses are enveloped, negative-sense, single-stranded RNA (ssRNA) viruses with a segmented genome. For instance, the influenza A virus (IAV) contains eight RNA segments encoding the RNA polymerase subunits [polymerase acidic protein (PA), polymerase basic protein 1 (PB1), polymerase basic protein 2 (PB2)], viral glycoproteins [hemagglutinin (HA), which facilitates viral entry; and neuraminidase (NA), which facilitates viral release], viral nucleoprotein (NP), matrix protein (M1), membrane protein (M2), and nonstructural protein 1 (NS1) (**Figure 1A**). IAV infects the cell through endosomal uptake and release, and the viral ribonucleoproteins (vRNPs) enter the nucleus for mRNA transcription and replication *via* a positive-sense complementary ribonucleoprotein (cRNP) intermediate. Viral mRNA is then converted into viral proteins in cytoplasm, which are complex into new virions and released from the cell (**Figure 1B**).

**Figure 2:**
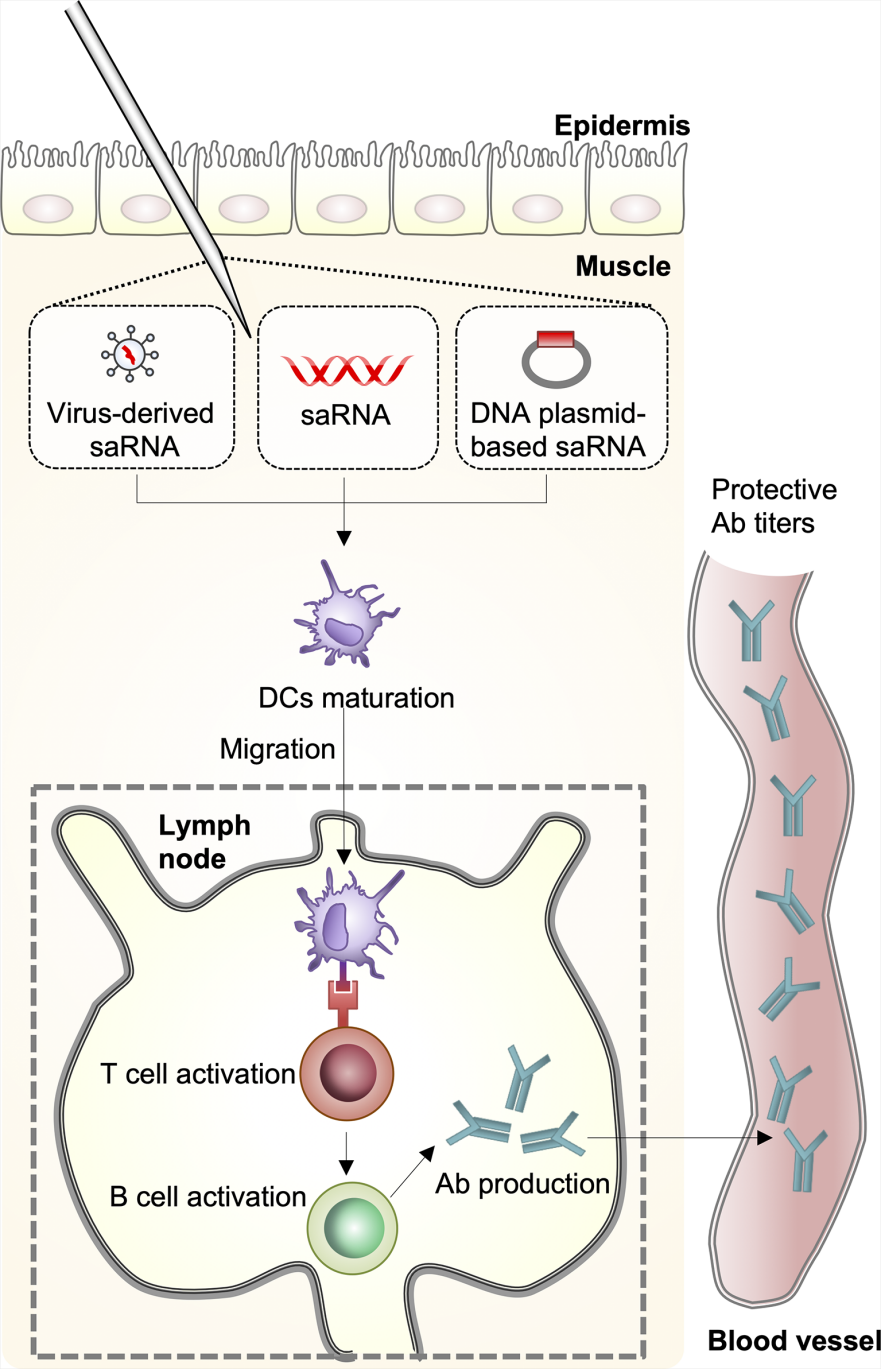
Host immune response against mRNA vaccines. A self-amplifying RNA (saRNA) vaccine can be delivered in the form of virus-like RNA particles, *in vitro* transcribed RNA, and plasmid DNA. Dendritic cells (DCs) recognize saRNA in the muscle and the cells are differentiated. The differentiated DCs function as antigen-presenting cells and migrate to the lymph nodes leading to activation of T- and B-cells for antibody production.

There are four distinct types of seasonal influenza viruses: types A, B, C, and D. Types A and B influenza viruses are currently co-circulating in the human population and cause seasonal epidemics: the H1N1 and the H3N2 subtypes of IAVs, and two divergent lineages (Yamagata lineage and Vitoria lineage) of the influenza B viruses (IBVs) (4). Through the coordination of World Health Organization Global Influenza Surveillance Network, seasonal influenza virus vaccines are designed annually and the formulations contain the two IAV strains and two IBV strains; however, the efficacy of seasonal influenza virus vaccines varies greatly each influenza season (5). Upon seasonal influenza vaccine injection, innate immune cells, including macrophages and dendritic cells (DCs) that are present in the muscle, cause an increase in the release of chemokines, which leads to the recruitment of more immune cells from the blood into the site of vaccination. The differentiated DCs act as antigen-presenting cells and migrate to the lymph nodes leading to the activation of T- and B-cells for the production of antibodies (6) (**Figure 2**). However, the vaccines induce narrow and strain-specific immunity that require constant updating (almost every year) due to the high frequency of point mutations, which ultimately represents a complex, expensive, and time-consuming process. In addition, various strains of influenza viruses, including avian and swine, have acquired the ability to grow efficiently in humans across species barriers from animal reservoirs and to disseminate between populations, posing a serious threat to humans. In addition to seasonal epidemics, influenza pandemics occur once every few decades and are caused by the swine-origin H1N1 IAVs, which have never circulated, and can spread rapidly across the human population. The H1N1 influenza virus pandemic in 1918 killed approximately 40 million people globally (7). Since then, pandemics have been caused by H2N2 in 1957, H3N2 in 1968, and again by H1N1pdm09 in 2009 (8). Considering seasonal influenza virus vaccines induce strain-specific immunity, which may not offer strong protection against infection by a pandemic-causing influenza virus, there is a need to develop novel influenza vaccines that induce broad immunity that is not focused on the corresponding vaccine strain (9).

Prior to the 1980s, inactivated and live-attenuated vaccines were established to protect humans against pathogenic microorganisms. Inactivated vaccines were manufactured by chemical or heat treatment, and live-attenuated vaccines were usually developed in animals or cell lines. The use of killed whole organism-based vaccines or live-attenuated vaccines had huge success in the reduction of viral infections including measles, mumps, rubella, and polio, and the eradication of smallpox infection (1). Despite the success, major concerns persist regarding conventional vaccination strategies. Infectious pathogens such as influenza virus are able to evade the adaptive immune response, and it is frequently difficult to develop live-attenuated or killed whole organism-based vaccines (10). In the case of the current influenza virus vaccine, the main challenge is its low effectiveness in the elderly (≥ 65 years old), who are more susceptible to influenza virus infection than children and adults (11). Such shortcomings of inactivated influenza virus vaccines are often attributed to mutations in surface antigens of the influenza virus. Other concerns regarding influenza virus live-attenuated vaccines include the potential to cause systemic inflammation and death, possibly due to back-mutations, acquisition of compensatory mutations, or recombination with circulating transmissible wild-type strains (12–14).

Generally, influenza virus infects the host *via* the nasal or oral cavities, where it makes the first contact with the respiratory epithelium, similar to live-attenuated influenza vaccines administered intranasally (15, 16). After infecting the respiratory epithelial cells, the virus or the intranasal influenza vaccine can induce both systemic and mucosal immune responses. The defense mechanisms of the innate immune cells, especially DCs, are a formidable barrier to the influenza virus. Particular immune systems exist at distinct mucosal surfaces to fighting invasion by influenza virus. The viral RNA that is present within infected cells is recognized as non-self by various pattern recognition receptors (PRRs), which leads to the release of proinflammatory cytokines, chemokines, and type I interferons (IFNs). Macrophages, pneumocytes, DCs, and plasmacytoid-DCs produce type I IFNs, which in turn stimulate the expression of IFN-stimulated genes (ISGs) in neighboring cells that induce antiviral states (17–19).

Unlike influenza virus infection or intranasal influenza vaccine, mRNA-based vaccines contain mRNAs that encode both the viral protein and immune-modulatory proteins as adjuvants, which enhance immunostimulatory properties. Upon mRNA entry into host cells, endosomal (e.g., TLR3, TLR7, and TLR8) and cytosolic innate immune sensors (e.g., RIG-I, PKR, NOD2, OAS, and MDA5) recognize mRNAs, including single-stranded RNA (ssRNA) and double-stranded RNA (dsRNA). Subsequently, the immune cells are activated and produce type I IFN and multiple inflammatory cytokines (20). However, single-stranded mRNAs in the vaccine are purified appropriately through *in vitro* transcription process to eliminate the interaction with innate immune sensors, which induce excessive secretion of type I IFN and its cellular dysfunction (20).

Furthermore, the adjuvant stimulates the innate immune response and drives antigen-specific T-cell responses without inducing systemic inflammation that could elicit severe side effects. In addition, injection site (e.g., subcutaneous, intramuscular, intradermal, and intravenous) and mRNA vaccine delivery formulations (e.g. lipid nanoparticle) influence the potency of the immune response (21). For instance, lipid nanoparticle carrier systems further protect mRNAs from nuclease, and can target delivery to lymphatics and promote protein translation in lymph nodes by intramuscular injection (20). In contrast to intranasal mRNA-mediated innate immune response, intramuscular mRNA will primarily be up taken by non-immune cells including local myocytes. Once in the lymph nodes, the lipid nanoparticle is eventually phagocytized by DCs, which consequently produce and present the antigen to T-cells to promote adaptive immune responses (22, 23). The capability of mRNA vaccines to induce the intracellular production of antigen proteins along with innate immune responses must prime both CD8^+^ and CD4^+^ T-cells to differentiate into effector and memory subsets for the protection from the infection (24).

The effective use of *in vitro* transcribed mRNA in mammals was first reported in 1990, with reporter gene mRNAs being injected into mice that induced the production of the target proteins (25). A subsequent study showed that administration of vasopressin-encoding mRNA in the hypothalamus could induce a physiological response in rats (26). However, such early promising results did not lead to significant investment for the development of mRNA vaccines because of anxieties associated with mRNA instability, severe innate immune responses, and ineffective *in vivo* delivery. Over the past decade, however, major technological innovations and investments in research have made mRNA a promising vaccination tool. The use of mRNA vaccines has several benefits over subunit, killed, and live-attenuated viruses. As mRNA is a non-infectious and non-integrating platform, there is no potential risk of infection or insertional mutagenesis. In addition, mRNA is degraded by normal cellular processes, and various modifications and delivery methods can modulate its half-life *in vivo* (27–29). Formulating mRNA into carrier molecules facilitates rapid uptake and expression in the cytoplasm, where it is stable and highly translatable (28, 30). Furthermore, mRNA vaccines have quick, inexpensive, and mountable manufacturing potential due to the high yield of *in vitro* transcription responses. Additionally, several elegant studies with non-human primate models of influenza mRNA vaccine have been carried out. After immunization of modified non-replicating mRNA encoding influenza H10 encapsulated in lipid nanoparticles in monkeys, protective levels of antibodies against hemagglutinin were dramatically increased, similar to observations following seasonal influenza vaccination in humans (31). Using a novel imaging technology (positron emission tomography–computed tomography, PET–CT), an mRNA vaccine labeled with a probe is trafficked to assess bio-distribution in monkeys, providing insights of the mechanisms of the innate and adaptive immune responses following vaccine administration on site and at the draining lymph node, which translates into protective immunity (32).

## Innate Immunity to Influenza Viruses

The IAV is recognized as foreign by PRRs including TLRs, RLRs, and NLRs, which leads to programmed cell death, secretion of type I IFNs, and proinflammatory cytokines, which induce excessive inflammation.

### TLR- and RLR-Associated Influenza Virus Recognition

TLR- and RLR-mediated signals for influenza viruses induce to the release of type I IFNs and stimulate the expression of ISGs by infected and nearby cells to induce an antiviral state (33–36). Moreover, TLR and RLR signals also induce the release of proinflammatory cytokines, such as interleukin (IL)-6 and IL-8 (37, 38).

TLR3 senses dsRNA in endosomes. Although the cells infected by influenza virus do not produce dsRNA, *Tlr3^−/−^* mice have a longer lifespan compared with wild-type mice after deadly influenza virus infection (38), suggesting that TLR3 recognizes an unidentified RNA structure following viral infection. Furthermore, *Tlr3^−/−^* mice produce normal antibodies, and CD4^+^ and CD8^+^ T-cell responses after influenza virus infection (33), suggesting that TLR3 is dispensable for generating T-cell immunity. In plasmacytoid-DCs, TLR7 recognizes the ssRNA genomes of influenza virus (34, 35). TLR7 signaling *via* adaptor myeloid differentiation primary response 88 (MYD88) leads to the activation of transcription factors including nuclear factor-κB (NF-κB) and IFN-regulatory factor 7 (IRF7), which induce the production of type I IFNs and proinflammatory cytokines (39–41).

RIG-I is a cytosolic PRR that generally recognizes 5’ppp-double-stranded RNA (dsRNA). RIG-I also senses the 5’ppp-viral ssRNA that is produced following influenza viral replication (42–44). After detection of influenza viral RNA, the helicase domain of RIG-I interacts to ATP, which facilitates conformational changes of the caspase-recruitment domains (CARD) to interact with signaling adaptor mitochondrial antiviral signaling protein (MAVS) for type I IFN production (45, 46).

### Inflammasome-Mediated Response to Influenza Virus

NLRs have an important role in antiviral immune response, inflammatory response, and cytokine induction. Proinflammatory signaling induces the recruitment of immune cell, such as macrophages and neutrophils, to eliminate pathogens and pathogen-derived molecules. IL-1β and IL-18 are key proinflammatory cytokines and are important for the protection against IAV infection (16, 47–49). Secretion of IL-1β and IL-18 requires the proteolytic maturation of pro-IL-1β and pro-IL-18, respectively, that is mediated by inflammasomes. Inflammasomes are multiprotein complexes consisting of caspase-1, ASC, and PRRs, such as NOD–like receptor family protein 3 (NLRP3) (16, 47–49). Upon viral ligand sensing, PRRs induce self-oligomerization and binds to the inflammasome adaptor protein ASC, which is composed of an N-terminal pyrin domain (PYD) and a C-terminal CARD. ASC oligomerizes *via* homotypic interactions of the PYD domain and then binds to caspase-1 *via* the CARD for the inflammasome formation, which ultimately induces inflammatory cell death.

In macrophages, influenza virus-induced NLRP3 inflammasome activation requires the Z-DNA binding protein 1 (ZBP1) to form the ZBP1–NLRP3 inflammasome (50, 51). When replicating, IAV generates Z-RNAs, which are transformed versions of an RNA double helix, that are sensed by ZBP1 and bind to the receptor interacting serine/threonine kinase 3 (RIPK3) and caspase-8 to activate the ZBP1–NLRP3 inflammasome (50–52). Recently, it has been shown that caspase-6 facilitates RHIM-dependent binding of RIPK3 with ZBP1, promoting ZBP1-mediated NLRP3 inflammasome activation (53). In respiratory epithelial cells, MxA functions as an inflammasome sensor that recognizes influenza viral protein NP and induces the release of IL-1β and IL-18 in an NLRP3-independent manner (15, 16).

NLRP3 and MxA inflammasome activation has been shown to be an important innate immune defense against influenza virus. After influenza virus strain A/Puerto Rico/8/34 (PR8) infection, NLRP3 was found to be important for the migration of leukocytes into the lungs and to protect the host from infection (47, 49). During influenza virus infection in human MxA transgenic mice, rapid activation of the MxA inflammasome in the respiratory epithelium showed to suppress viral spreading from the bronchioles to the distal alveolar regions (16). The results of such studies suggest that optimal inflammasome activation is beneficial for the host; however, abnormal activation can cause to harmful outcomes.

## Adaptive Immunity to Influenza Virus

Innate and adaptive immune responses significantly protects the host from influenza viruses and are important for the production of strong antibody responses. After influenza virus infection, the innate immune system is critical for recognizing and removing vaccinated cells while also coordinating an adaptive immunity through an antigen-specific antibody reaction and providing long-term protection from the viral infection.

Upon recognition of viral antigens and interaction with cognate CD4^+^ T-cells, naïve B-cells are activated (54). Some of the activated B-cells quickly differentiate into short-lived plasmablasts. Although other activated B-cells migrate to the follicles of secondary lymphoid tissues and undergo a germinal center reaction, the plasmablasts produce the first wave of virus-specific antibodies in humans. If plasmablasts are derived from memory B-cells, the numbers of plasmablasts peak in the periphery at about 7 days post-infection (55). A small number of activated B-cells, however, will only differentiate into long-lived plasma cells, which migrate and resides in the bone marrow to produce antibodies, which provide the long-term serum antibody level and are associated with the defense against pathogen infection and disease. Another portion of the primarily activated B-cells differentiate into memory B-cells (56, 57), which do not release antibodies and remain in the periphery for immune surveillance, but they are long-lived and can be reactivated to become plasmablasts during infection for the production of new antibodies and further memory B-cells (55). Overall, the adaptive immunity-derived antibody response to influenza virus infection is relatively broad and long-lived; however, influenza viruses can deviate from the adaptive immune responses over time owing to their high mutation rates and antigenic flexibility. Further studies of how natural viral infection induces long-lived immune responses are required for the development of next-generation influenza virus vaccines.

## Conventional Seasonal Influenza Virus Vaccines

Seasonal influenza virus vaccines are produced using egg-, cell-, and protein-based technologies, and the entire processes typically take 6~8 months. Influenza vaccines against IAVs and IBVs were invented in the 1940s and such whole-virus inactivated vaccines were generated in embryonated chicken eggs that consisted of crudely purified whole virus inactivated with formalin and phenylmercuric nitrate (58, 59). In 2012, vaccine-containing cell-based virus emerged as an alternative method for producing inactivated and live-attenuated vaccines (60). The efficacy of trivalent inactivated and live-attenuated influenza vaccines is approximately 65% and 83% in adults and children, respectively (61). Live-attenuated influenza virus vaccines that are used are usually cold-adapted and temperature-sensitive, and efficiently replicate in the upper but not the lower respiratory tract (62–64). Collectively, such studies indicate that seasonal influenza virus vaccines present good protection against influenza virus infection.

However, there are also negative reports on such vaccines. A vaccine efficacy of 75% in adults declines suddenly in the elderly, who are more susceptible to influenza virus infection (65, 66). In addition, mismatches between vaccine and circulating strains sometimes occur and are generally associated to lower vaccine efficiency (67). Based on US virologic surveillance and US Influenza Vaccine Effectiveness (Flu VE) Network data, the estimated vaccine effectiveness (VE) against IAV or IBV decreased to 29% in all ages during the 2019–2020 influenza season (average VE in 2010–2018 was 44.1%) due to the spread of antigenically drifted IAV (H3N2), which has raised concerns about vaccine strain selection (68) (https://www.cdc.gov/flu/vaccines-work/past-seasons-estimates.html). Furthermore, the duration of protection is occasionally short (69, 70). To induce stronger, more sustained immune responses in the elderly, high-dose vaccines or advanced adjuvant vaccines including MF59 and AS03, have been tested. The doses in high-dose vaccines are 4-fold those in trivalent inactivated vaccines, which could induce higher amounts of antibodies than standard dose vaccines (71). MF59-adjuvanted seasonal vaccines have been licensed and marketed in more than 25 countries for the elderly population (72, 73). AS03-adjuvanted influenza virus vaccines are also under consideration for use in the elderly population (74).

## Conventional Pandemic Influenza Virus Vaccines

IAVs are typically transmitted within one animal species but sometimes they can cross over and cause illness in another species. In the latter situation, more significant genetic changes are exhibited than in circulating human seasonal IAV. If the novel IAV is infectious and spreads easily in humans, it is considered an influenza virus pandemic. Because most of the human population has no or only limited natural immunity to novel influenza virus strains, pandemic IAV vaccine candidates have been developed using a range of production platforms. The inactivated split influenza virus vaccine platform with an adjuvant has advanced the furthest, and other platforms, including nucleic acid (DNA and mRNA), vector, recombinant protein (VLP; virus-like particle), and live virus are still under development (75). H5N1 avian influenza virus vaccine was the first U.S. FDA-approved vaccine for the human population because concerns were raised with regard to the potential of the highly pathogenic H5N1 virus to cause an influenza pandemic (76, 77).

One of the challenges in influenza pandemic management is the global vaccine production capacity within several months following the emergence of an outbreak. In the case of vaccines against H5N1 strains, the seed strains were generated using reverse genetics to remove the multi-basic cleavage site of the HA and to alter the backbone to that of a high-growth PR8 H1N1 strain, which exhibits relatively less pathogenicity (77). Such modifications facilitate the production of safer vaccine strains and at high quantities, because highly pathogenic IAVs frequently kill embryonated eggs, resulting in low production (77). A number of the H5N1 and H7N9 vaccines have been tested in humans and a high antigen dose or the use of an adjuvant is necessary to induce reliable hemagglutination inhibition titer (77, 78). A clinical trial of an H7N9 vaccine yielded an efficacy of approximately 60% despite the use of an adjuvant (79), indicating that adjuvants and multiple vaccinations are required for achieving sufficient vaccine efficiency.

## Messenger RNA Vaccine Strategies Against Influenza Virus

mRNA vaccines are extensively characterized by experimental approaches that improve mRNA stability and delivery, and protein production. Such approaches involves the development of nanoparticle transport techniques that stabilize mRNA, enhance cellular uptake, and improve biological availability when mRNA enters the cell. A clear advantage of mRNA vaccines is that it does not need to enter the nucleus to promote antigen expression.

### Self-Amplifying mRNA Vaccines Against Influenza Virus

**Figure 3:**
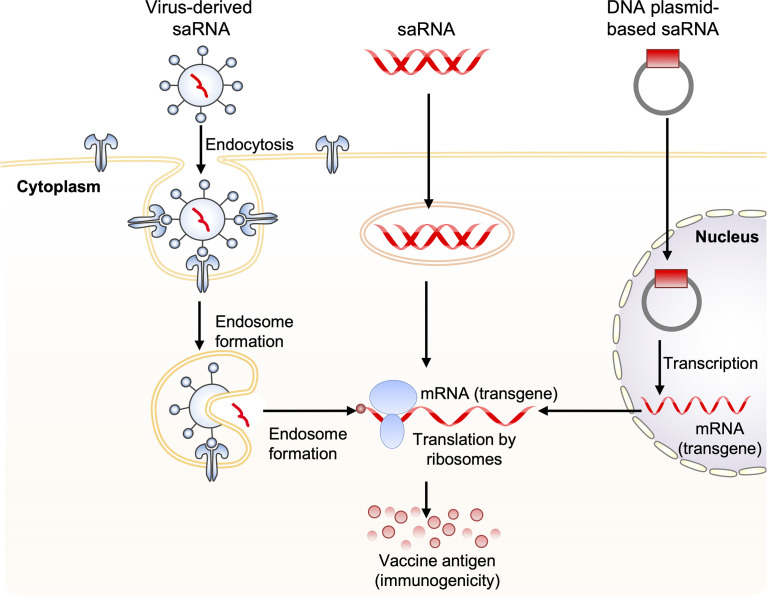
Obtaining antigen expression by self-amplifying RNA vaccination. Three routes for the delivery of self-amplifying RNA (saRNA) are shown. These include: 1) virus-like RNA particles that deliver saRNA to the cytoplasm by receptor-mediated endocytosis, 2) direct delivery of *in vitro* transcribed replicon saRNA to cells either in saline or in synthetic formulations, and 3) plasmid DNA carrying replicase genes and the transgene into the nucleus, where it is transcribed, generating replicon saRNA, which is then transported to the cytoplasm. The three saRNA vaccination routes finally generate messenger RNA (mRNA), which is translated *via* ribosomes to produce vaccine antigen.

A self-amplifying RNA (saRNA) vaccine can be carried in the form of plasmid DNA, virus-like RNA particles, and *in vitro* transcribed RNA (80) (**Figure 3**). DNA plasmid-based saRNA vaccines combine the advantages of a more stable DNA nucleic acid product with greater levels of antigen expression. saRNA vaccines have elicited strong immune responses in preclinical models (81). saRNA vaccines are derived from the genome backbone of an alphavirus, in which the genes encoding the viral RNA replication machinery are intact (82).

There are several reports on the use of saRNA vaccines against influenza virus. Immunization with 10 mg of saRNA vaccine encoding PR8 H1N1 IAV HA produced antibody responses and protection from lethal homologous viral challenge in mice (83). Another study showed development of saRNA vaccines where the A/California/07/2009 (H1N1) or A/Shanghai/2/2013 (H7N9) IAV HA-encoding saRNA was formulated in lipid nanoparticles, and small doses induced protective levels of hemagglutination inhibition titers after two intramuscular injections in mice (84). Consistent with this finding, another group showed that intramuscular administration of 0.1 or 0.2 mg of PR8 H1N1 IAV NP, M1, or combined NP+M1 self-amplifying RNA-lipid nanoparticle vaccines resulted in strong antigen-specific T-cell responses and protection from homologous viral infection (85). IAV HA and NP replicon RNA complexed with chitosan-containing lipid nanoparticles or polyethylenimine (PEI) has elicited T- and B-cell immune responses in mice after subcutaneous delivery (86, 87). A recent study revealed a delivery platform consisting of a chemically modified, ionizable dendrimer complexed into lipid nanoparticles. Using this platform, intramuscular delivery of RNA replicons encoding A/WSN/33 (H1N1) IAV HA successfully protected mice against lethal homologous virus challenge (88). A more recent study showed a direct comparison of the immune response and protective efficacy after self-replicating mRNA and non-replicating mRNA immunization in mice (80). Animals were intramuscularly immunized two times with increasing doses of PR8 H1N1 IAV HA-encoding unformulated self-replicating mRNA or unmodified non-replicating mRNA and challenged eight weeks after the first vaccination. Both platforms induced protection against infection with the homologous influenza virus (80).

### Non-Replicating Influenza Virus mRNA Vaccines

Non-replicating mRNA vaccines can be made with the incorporation of various modified nucleosides, and much effort has been invested in the development of directly injectable non-replicating mRNA vaccines. There are several studies of non-replicating mRNA vaccines against influenza virus. Administration of IAV NP-encoding mRNA complexed in liposomes induced cytotoxic T-cell responses in mice (89). Intradermally immunized mice, ferrets, and pigs with various IAV HA-, NP-, and NA-encoding RNActive vaccines produced protective immune responses after a single immunization (90). Intravenous immunizations in mice using PR8 H1N1 IAV HA-encoding unmodified mRNA-lipid complexes showed elevation of T-cell activation after administration of a single dose (91). Together, it will be important to determine if and how these findings translate to clinical trials and vaccination.

## Innate Immunity to mRNA Vaccines

The innate immune system acts as the first line of host immune response against mRNA vaccination. During an mRNA vaccination, the mRNA can be recognized by various PRRs, including TLRs, RLRs, and NLRs, for the production of IFNs and proinflammatory cytokines. Prototypically, IFNs and proinflammatory cytokines are largely beneficial to the host and trigger induction of IFN-inducible genes and the infiltration of immune cells to remove vaccinated cells. However, dysregulated IFNs and proinflammatory cytokines drive inhibition of efficacy of mRNA vaccines, detrimental systemic hyperinflammation, and tissue damage. Therefore, appropriate purification of *in vitro* transcribed mRNA is critical for maximizing immunogen production and avoiding undesired innate immune activation.

TLR3 senses dsRNA longer than 45 base pairs and those derived from ssRNA forming secondary structures or from viral replication intermediates. TLR7 can recognize both dsRNA and ssRNA, whereas TLR8 only senses ssRNA (92). The activation of TLR7 can stimulate antigen presentation, cytokine release, and induce B-cell responses (93). In particular, RIG-I senses ssRNA and dsRNA bearing a 5′-triphosphate and stimulates IFN production (44, 94).

**Figure 4:**
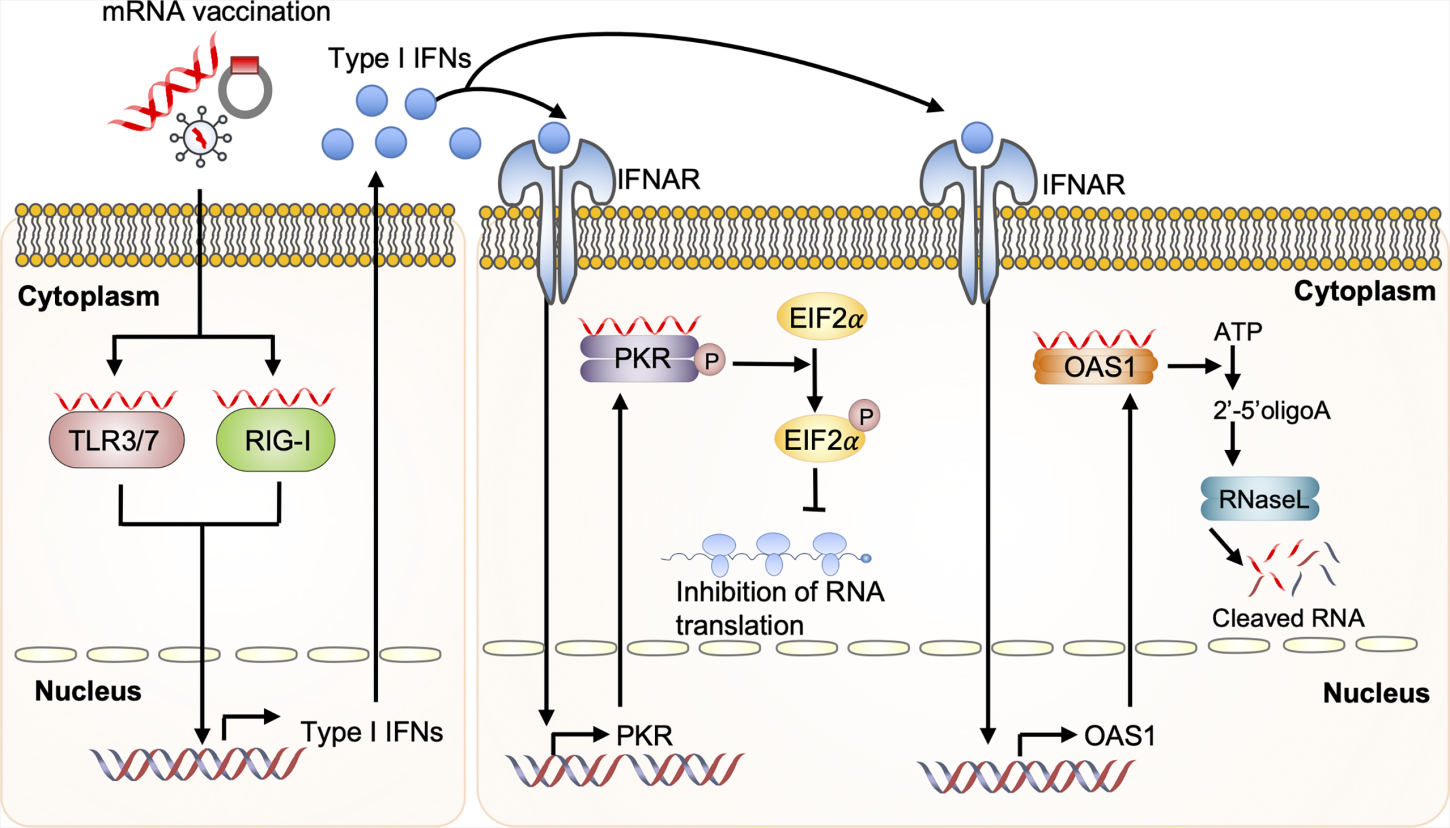
Dysregulated innate immunity prevents mRNA vaccine efficacy Upon mRNA vaccination, incoming self-amplifying RNA (saRNA) is recognized by the Toll-like receptor (TLR) 3, TLR7, and retinoic acid-inducible gene I (RIG-I), which promotes their downstream signaling and consequent production of type I interferon (IFN). Recognition of *in vitro* transcribed mRNA contaminated with dsRNA causes rapid type I IFN production, which induces the expression and activation of protein kinase R (PKR) and 2′-5′-oligoadenylate synthetase (OAS). This will inhibit the translation and induce the degradation of cellular mRNA, ribosomal RNA, and *in vitro* transcribed mRNA. EIF2a, eukaryotic initiation factor 2a; IFNAR, interferon-alpha receptor.

mRNA vaccine-derived IFN production *via* RNA sensors is dependent on the quality of *in vitro* transcribed mRNA, the delivery vehicle, and the administration route. Recognition of *in vitro* transcribed mRNA contaminated with dsRNA cause rapid type I IFN induction, which upregulates the expression and activation of protein kinase R (PKR) and 2′-5′-oligoadenylate synthetase (OAS). This leads to the inhibition of translation and the degradation of cellular mRNA, ribosomal RNA, and *in vitro* transcribed mRNA (95–97) (**Figure 4**). Contaminating dsRNA can be efficiently removed from *in vitro* transcribed mRNA by chromatographic methods including reverse-phase fast protein liquid chromatography or high-performance liquid chromatography. Purification by these methods has been shown to increase antigen protein production from *in vitro* transcribed mRNA by up to about 1000-fold in human DCs (97). In addition to dsRNA contaminants, single-stranded mRNA molecules are recognized as a PAMP and detected by TLR7 and TLR8, resulting in type I IFN production and decreased antigen protein production from *in vitro* transcribed mRNA (98–100).

Given that reducing type I IFN signaling is critical for mRNA vaccine strategy, several studies revealed the development of modified mRNA that reduced type I IFN signaling. Incorporation of naturally occurring chemically modified nucleosides, including pseudouridine and 1-methylpseudouridine, prevented the activation of TLR7, TLR8, and other innate immune sensors, thereby reducing type I IFN signaling (27, 101–104). Another study also showed that nucleoside-modified mRNA translated better compared with that of unmodified mRNA *in vitro*, especially in primary DCs, and *in vivo* in mice (27, 103). Together, the balance between mRNA vaccines and the innate immune response is critical for potential vaccine approaches; this balance must be finely controlled to reduce excessive innate immune responses while retaining maximal immunogen production in DCs.

Note, that while the inflammasome-dependent release of IL-1b and IL-18 triggers further production in DCs for the induction of influenza virus-specific CD8^+^ T-cell priming (49, 105), whether *in vitro* transcribed mRNA can be recognized by inflammasome sensors remains largely unknown. Future studies are needed to fully examine the factors required for the inflammasome-driven adaptive immune response.

## mRNA-Based Influenza Virus Vaccine Development

mRNA vaccines have lately attracted substantial attention, involved massive academic and industrial investment, and biotechnology companies have succeeded in raising capital for the development of innovative RNA vaccines. The first directly injectable influenza RNA vaccine against H10N8 and H7N9 was developed by scientists at Moderna Therapeutics (USA), who demonstrated that it was possible to induce protective immunogenicity with acceptable tolerability profiles (106, 107). For the first human seasonal influenza virus RNA vaccine trial, CureVac AG company (Germany) developed a sequence-engineered mRNA-lipid nanoparticle strategy that enables an extraordinary level of *in vivo* protein translation without the use of modified nucleosides (29). Using this strategy, a recent study demonstrated that a single intramuscular immunization with 10 μg of A/Netherlands/602/2009 (H1N1) HA-encoding mRNA-lipid nanoparticles induce hemagglutination inhibition titers in the protective range (≥1:40) in non-human primates. Moreover, inoculation of a second dose effectively boosted immune responses and resulted in hemagglutination inhibition titers ≥1:160 for over 1 year in all vaccinated animals (108). CureVac AG scientists provided evidence that two immunizations with mRNA-lipid nanoparticles encoding for HA from A/Hong Kong/4801/2014 (H3N2) induced stronger T- and B-cell immune responses than the licensed MF59-adjuvanted trivalent inactivated IAV vaccine.

## Limitations of mRNA Vaccine

mRNA vaccine-associated hypersensitivity reactions are not often observed. Similarly, severe acute-onset, presumably immunoglobulin-E (IgE)- or immunoglobulin-G (IgG)-mediated, and complement-mediated anaphylactic or severe delayed-onset T-cell-mediated systemic responses are considered very rare. Hypersensitivity to the active antigen of the vaccine can also be developed. Acute hypersensitivity responses following vaccination include self-limited local side effects and systemic responses can range from urticaria/angioedema to full-scale anaphylaxis with multisystem involvement (109).

Anaphylaxis is a rare life-threatening allergic reaction that usually occurs within minutes to hours after vaccination (109). Anaphylaxis induced by vaccines is generally rare. The coronavirus disease 2019 (COVID-19) pandemic highlighted the need for robust vaccine production *via* mRNA vaccines derived in lipid nanoparticles. Unexpectedly, there are fewer serious allergic reactions due to public vaccines and, as a result, there are considerable public concerns centered on atopic individuals. Previous research on the immune mechanism of vaccine-related anaphylaxis has focused on the presence of gelatin, latex, and egg proteins, and more recently on polysorbate 80, a widely used surfactant present in many vaccines (110). However, all of the above-mentioned excipients were not included in the Pfizer-BioNTech COVID-19 mRNA vaccine and no cases of anaphylaxis were observed in large-scale phase 2/3 clinical trials; thus, these events are unexpected (111, 112). Hence, occurrence of anaphylaxis upon initial exposure to the COVID-19 vaccine refers to pre-existing antibody-mediated immunity (allergy) or a pseudo-allergic reaction unrelated to previous exposure.

Anaphylaxis associated with known allergens is best understood through the classical paradigm of crosslinked IgE bound to fragment crystallizable region (Fc) ϵ receptors of mast cells and basophils, but nonclassical pathways including antibody-dependent activation of complement or IgG-associated mast cell/granulocyte/platelet/basophil-mediated mast cell/granulocyte/platelet/basophil activation *via* Fcγ receptors has been described in animal models and in human allergic responses to drugs (113–117). Unfortunately, information on the potential use of vaccines for testing to confirm the pathological etiology or predict reactivity risk remain scarce.

A rapid and thorough study-based evaluation of patients who have experienced anaphylactic vaccine reactions and prospective clinical trials in individuals at risk are requested to address these concerns during the public health crisis.

## Conclusions and Future Research Directions

We have highlighted the innate immune system response during IAV mRNA vaccination. Optimal innate immune sensing and inflammatory cytokine release are essential for T- and B-cell immune responses. However, excessive host innate immune responses possibly lead to cytokine storms and/or tissue damage (118, 119), inhibiting the efficacy of influenza virus mRNA vaccines. Therefore, mRNA-induced innate immunity must be controlled to reduce excessive inflammation while retaining antibody production.

Over the past decade, there has been a clear breakthrough in the field of influenza virus mRNA vaccines, demonstrating proof-of-concept in both preclinical and clinical settings (20). However, reducing the innate immune sensing of mRNA and maximizing translatability of the promising animal data to humans remains challenging. Further clinical trials examining the long-term safety and immunogenicity are required to evaluate the impact of RNA vaccines on the influenza virus vaccine field.

Currently, the COVID-19 pandemic continues to spread globally, urgently requiring effective vaccines to fight it. FDA-approved mRNA vaccines are greatly effective in working-age adults at preventing SARS-CoV-2 infection, with the vaccines attenuating the viral RNA load, risk of febrile symptoms, and duration of illness among those who have breakthrough infection despite vaccination (120). The future of mRNA vaccine field is potential, and the clinical data and resources provided by the associated companies and other academic institutions are likely to significantly build on and strengthen basic research into mRNA-based vaccines.

## Author Contributions

SL and J-HR conceived the outline of the manuscript. SL and J-HR. wrote the manuscript. SL and J-HR critically revised and approved the final version of the manuscript. All authors contributed to the article and approved the submitted version.

## Funding

This research was supported in part by grants-in-aid from the Ministry of Education, Culture, Sports, Science, and Technology of Japan (19K16665 to SL).

## Author Disclaimer

The content is solely the responsibility of the authors and does not necessarily represent the official views of the National Institutes of Health.

## Conflict of Interest

Author J-HR was employed by company Biorchestra Co.

The remaining authors declare that the research was conducted in the absence of any commercial or financial relationships that could be construed as a potential conflict of interest.

## Publisher’s Note

All claims expressed in this article are solely those of the authors and do not necessarily represent those of their affiliated organizations, or those of the publisher, the editors and the reviewers. Any product that may be evaluated in this article, or claim that may be made by its manufacturer, is not guaranteed or endorsed by the publisher.
